# Silver Sutures

**Published:** 1858-09

**Authors:** 


ART. V.—*Silver Sutures. The Anniversary Discourse before the New York Academy of Medicine. Delivered in the New Building of the Historical Society, on the* 18*th November,* 1857. By J. MARION SIMS, M. D., Surgeon to the Woman's Hospital. (Published by order of the Academy.) New York: SAMUEL S. & WILLIAM WOOD, 389 Broadway. 1858.

Among the medical societies which have been instituted for the advancement of science, the New York Academy of Medicine occupies, perhaps, the most dignified position, and anything which emanates from that learned body, carries with it a weight which is recognized by the profession of this and other countries. In addition to this passport to favor, the reputation which Dr. Sims has attained by his novel and successful treatment of the diseases arising from difficult or obstructed labor, led us to expect a valuable contribution to science, when we read that he has chosen this interesting theme for the subject of his anniversary discourse. This discourse, therefore, will be, or has been, very generally read, and demands attention from the journalist, whose duty it is to keep the profession *au courant* in matters of scientific interest.

We have read, and carefully read the paper of Dr. Sims, and in spite of the endorsement of the Academy and the reputation of the author, we would have been tempted to shrink from the disagreeable task which we have before us, were it not for the malignant and uncalled for attack upon a fellow-worker in this department of reparative surgery.

This is the only part of the discourse which inspires in us anything but sympathy for the ridiculous and pitiable position in which the bombastic inflation of the author has led him to place himself before the profession; and in pity then, we would have left the criticism of his paper to its readers; and those who were so unfortunate as not to have seen it, we would have left in ignorance of the extent to which prosperity could annihilate the common sense of an intelligent man.

We have spoken strongly, and we intended to speak strongly; but our editorial conscience will not permit us to pass over such a production, containing as it does gross and unfounded imputations against the character of one who has done much for science, and whose reputation, every one acquainted with the literature of vesico-vaginal fistula must respect. We shall speak in its proper place, of the charges which are brought by the author against Dr. Bozeman, and must now say something in justification of the remarks which we have felt called upon to make.

The oration commences, as many orations have commenced before, with an expression of diffidence in following in the footsteps of those to whom this duty had been assigned on the preceding anniversaries. Then follows a laudation of his predecessors, which, though we all know it to be most amply merited, could not have failed to have called a painful blush to their cheeks. After which he proceeds to the proper subject of his discourse, "the use of silver sutures in surgery."

All surgeons are familiar with the operation described by Dr. Sims in the American Journal of Medical Sciences for January, 1852. Could not Dr. Sims have been satisfied with the favor with which his discovery was received by the profession? Kollock, the author of an able report on this subject, Wood, Mettauer, Williams, of this country, and Isaac Baker Brown, Druit and Spenser Wells, of Great Britain, with many other eminent surgeons, bear testimony to its excellence, and have used it with success. Baker Brown, who is one of the highest English authorities on this subject, in his work on "Surgical Diseases of Women," places the operation described by Dr. Sims before *all others.*

We all give honor to Dr. Sims, and claim for our country the credit of having taught the world a mode of relief for these disgusting maladies, after such men as Desault, Roux, Dieffenbach, Baker Brown, and the host of eminent surgeons, have for centuries acknowledged that vesico-vaginal fistula was almost without a remedy. Men cannot but admire the talent and industry which have wrought such an improvement in any department of science, and especially one which brings relief to the horrible sufferings which so loathesome a disease entails upon its victims; but his admirers cannot be the less mortified that he has descended so far from what they would have expected, and must deplore the ridiculous egotism and intolerance which indicate so clearly that, while Dr. Sims had the strength to achieve a great work, he is too weak to bear the credit and prosperity which are the result of his labors.

The operation of Dr. Sims we can describe in a few words. The edges of the fistula are to be freely pared, a point to which he properly attaches a great deal of importance, and brought together by sutures of fine silver wire, fastened to leaden clamps. The ends of the wires are made to perforate the clamps, and are held in their places by perforated shot compressed upon them. The whole is then allowed to remain until union by the first intention becomes firm, and is then removed by clipping off the shot with cutting pliers.

We will now quote from the oration the passage which refers to Dr. Bozeman's alleged piracy of his laurels:

"The city of Montgomery, Alabama, was the theatre of my early operations. Bad health compelled me to leave them in 1853. I then gave Dr. Bozeman a partnership in business, and indoctrinated him in my peculiar method of operating for vesico-vaginal fistula, instructing him in my various modes of using silver wire as a suture, not only in this class of affection, but in general surgery. Not understanding its principle of action, and therefore failing in its practical application, he was quite disheartened with his ill-success, when by mere accident he fell upon a plan of fastening the wire, and so modifying my method, that in awkward and inexperienced hands it became easier of application. Instead of passing the wires through the leaden bars on each side of the fistula, he passed them through a concave disk, or 'button,' which rests upon the surface of the parts to be united.

"Notwithstanding the fact that the doctor lived in Montgomery for years, without any professional position till I gave it to him, that he is indebted to me for what he never could have obtained without my aid, he appropriates to himself every step of the operation that resulted from my own individual and unaided efforts—even my silver wire and perforated shot, the only things of any real value whatever, and publishes it as his operation by a 'new mode of suture,' making strenuous efforts to place my labors entirely in the back ground.

"I do not complain of modification, but I do complain of a disingenuousness which would be dishonorable, even under widely different circumstances."

Here, in the first place, the author states that "not understanding its principle of action, and therefore failing in its practical application, he was quite disheartened with his ill-success." It does not seem to us possible that any person of common intelligence could mistake the "principle of action" of the clamp suture; and it also seems to us that it was because Dr. Bozeman understood fully the "principle of action" of the new method, and appreciated its deficiencies, that he was led to invent the button modification. By referring to an able review of Sims, Bozeman, and Kollock, in the October number of the American Journal of Medical Sciences, 1857, by Dr. Storer, it will be seen that the profession at large have "*not understood its principle of action;*" that the wires will cut themselves out in certain instances; and Dr. Sims himself acknowledges that

"The clamps, burrowing in the vaginal surface, leave a deep sulcus by the side of the new cicatrix, which, when they are removed too soon, fill up by granulation. It is a law of all granulating wounds, to contract as they heal, and this contraction on each side of the new cicatrix is often sufficient to pull it gradually apart. Accidents of this sort have happened repeatedly in my hands from a too early removal of the suture apparatus. Great judgment, which experience alone can give, is necessary to determine the length of time that the sutures ought to remain intact, for no positive rules can be laid down that will answer invariably in every case. I have also seen serious mischief result from leaving the clamps too long embedded in the parts. Their burrowing and ulceration may extend entirely through the vagino-vesical structure, thereby substituting new fistulous opening for the original one. This complication is by no means incurable, but only prolongs the treatment and postpones ultimate success. In two or three instances I have witnessed a still more serious accident from an undue pressure of the clamps, viz., a strangulation of the enclosed fistulous edges which unfortunately resulted in a sloughing of the tumefied parts, and a consequent enlargement of the opening.*

Other difficulties have been shown to occur, by Dr. Kollock, in his report before the State Medical Society of Georgia; and Dr. Storer states that Colles, of Dublin, and Spencer Wells, of London, have endeavored to devise means of avoiding these accidents. Their plans, indeed, have been published, but Dr. Storer gives the palm to Dr. Bozeman, *who had not the intelligence to understand the "principle of action" of Dr. Sims' method*, and who repaid the benefits which Dr. Sims' had heaped upon him, by daring to give to science an improvement of the clamp suture "so that in awkward and inexperienced hands" the operation could be made successful.

"It is to Dr. Bozeman, of Alabama, to whom it accidentally suggested itself, that we are indebted for the long looked for discovery now known as the button suture. His first paper was published in the spring of 1856, and he has lately made known the results of a more extended experience, by diagrams, accurate descriptions, an elaborate classification of all possible vari-

* Dr. Sims' article in the American Journal of Medical Sciences, January, 1852, page 70.

eties of fistula, and directions for perfectly adapting his apparatus to each and every one of them."*

The same reviewer states that Dr. Sims has judiciously combined the stitch of Hayward, and the metal thread of Mettaner; which suggestions he gives not the slightest credit to Dr. Hayward, and merely mentions that lead sutures had been used by Mettaner and Dieffenbach; he had tried them, "but fortunately for science, the clumsy lead wire was unsuccessful in my hands." Yet, when Dr. Bozeman publishes his improvement, the only original point which he claims, being the button, because he does not state in so many words that he uses the silver wire and perforated shot, *recommended by Dr. Sims*, a fact which is sufficiently well known to every surgeon, he is accused of the blackest literary piracy, and every epithet is heaped upon him which could be tolerated by the august body before which the oration was delivered. Dr. Sims then reports seven cases in which he has tried the button suture of Dr. Bozeman, and which seemed to him particularly favorable for the operation, six of which was unsuccessful. The *animus* with which the improvement of Dr. Bozeman is discussed, would be a sufficient argument against the fairness of this test, if we did not have *twenty-four* operations reported by Dr. Bozeman, in which he had used the button suture, which were successful in *twenty-two* instances, and the two which were not entirely successful were only partial failures. In addition to this, we have the testimony of such men as Gross, Storer, Kollock and others, in favor of the superiority of the button suture.

In this connection, though we have already noticed Dr. Bozeman's article in a former number of this Journal, we cannot refrain from speaking of a surgical achievement, not even mentioned by Dr. Sims, which was conceived, but not executed, by Jobert; that of paring and suturizing the cervix uteri. To Dr. Bozeman alone, is the credit due, of having performed successfully this novel and bold exploit.

We conceive that we have said enough, and more than enough, to defend Dr. Bozeman from the unprovoked assault of Dr. Sims, and will now endeavor to follow the author through the remainder of his discourse, as closely as our limits will permit.

The style in which our author indulges is unheard of in scientific literature; and though it may fix the attention more closely, from its novelty, yet we should be sorry to see the example followed by others who may have

* Review by Dr. Storer, loc' citat'.

anything to present to the profession. The simplicity of the truly scientific writer gives an additional charm to a valuable production. Such a charm is not lent to the paper before us by the flaming capitals which head each page, as "EFFORTS TO SUPPLANT AUTHOR," "PROVIDENTIAL INCIDENT," "WOMAN'S MORAL COURAGE," "NEW HOPES SUDDENLY INSPIRED," "PERSONAL NARRATIVE," etc. These striking headings are the first that meet the eye of the reader as he opens the pamphlet, and are a truthful indication of what he finds beneath them. The most "thrilling" story in a Sunday newspaper could hardly compare, in graphic description, with some passages of Dr. Sims' oration. For example, take the following, which is on the page headed by "NEW HOPES SUDDENLY INSPIRED."

"*Full of thought I hurried home—and the patient, (with vesico-vaginal fistula,) who was to have left the next day, was placed in the position described,* with an assistant on each side to elevate and retract the nates. I cannot, nor is it needful to describe my emotions, when the air rushed in and dilated the vagina to its utmost capacity, whereby the whole surface was seen at one view, for the first time by any mortal man. With this sudden flash of light, with the fistulous opening seen in its proper relations, seemingly without any appreciable process of ratiocination, all the principles of the operation were presented to my mind as clearly as at this time. And thus, in a moment, in the twinkling of an eye, new hopes and new aspirations filled my soul, for a flood of dazzling light had suddenly burst upon my enraptured vision, and I saw in the distance the great and glorious triumph that awaited determined and persevering effort. I thought only of relieving the loveliest of God's creatures of one of the most loathsome maladies,*" *etc. etc. etc.*

The author then goes on with his "personal narrative," relating how he was occupied from the 9th of December, 1845, to the 10th of January, 1846, in procuring the proper instruments; and on the latter date, the first operation was performed. The first was a simple case, but what was his surprise and mortification to find it a failure. These failures were repeated until all his professional friends, who had previously encouraged him in his efforts, deserted him. We have not time to follow the orator through all his sensations, etc., until he discovered the application of the perforated shot and the silver wire; suffice it to say that he finally made the operation in the manner we have hastily described in the beginning of this article, and "having contracted the chronic disease of a warm climate which is almost universally fatal, and struggled hard for more than two years, and, as it seemed,

* On her elbows and knees, with the pelvis elevated and the thorax depressed.—ED.

hopelessly against my fate, thus seeing death was inevitable, and fearing that I might die without the world's reaping the benefit of my labors, I determined to give my experience, crude as it was, to the profession that I loved so much," like Napoleon the First, "Je désire que mes cendres reposent sur les bords de la Seine, au milieu de ce peuple Français que j'ai tant aimé."*

Here we have depicted the trials and persecutions of a Jenner or a Harvey; the hopes, fears, and labors which a vivid imagination would connect with the establishment of a startling and almost incredible discovery, with a dash of the "Little Corsican" worked in at the end. But no, Dr. Sims has not been oppressed! He has not been persecuted! His discovery has met with a favor which is not often conceded, immediately, to a new thing, and while he has shown that he could conquer adversity, he is not proof against the more subtle dangers of prosperity.

But we cannot close this review without again expressing our sense of the value of the labors of Dr. Sims, which we have no intention to underrate, though we deplore the errors of style into which he has fallen, and are compelled to censure his attack upon his more unassuming fellow-laborer, who, we are confident, has made an important improvement in his admirable operation. As we have before remarked, Dr. Sims has all the credit, as the first man who treated vesico-vaginal fistula with success, and it is not usually given to one man both to originate and perfect an important invention. That Dr. Bozeman has made an improvement by the use of the button, we have the testimony of the highest authorities in this country; but this improvement in no wise detracts from the merit of the original invention. The strictures which we have made, were actuated by no prejudice or personal feeling, and we have simply said what we thought to be the right.

---

* I desire that my ashes may repose on the banks of the Seine, in the midst of the French people I have loved so much.

